# Validation of a Monte Carlo simulation for Microbeam Radiation Therapy on the Imaging and Medical Beamline at the Australian Synchrotron

**DOI:** 10.1038/s41598-019-53991-9

**Published:** 2019-11-27

**Authors:** Andrew Dipuglia, Matthew Cameron, Jeremy A. Davis, Iwan M. Cornelius, Andrew W. Stevenson, Anatoly B. Rosenfeld, Marco Petasecca, Stéphanie Corde, Susanna Guatelli, Michael L. F. Lerch

**Affiliations:** 1 Centre for Medical and Radiation Physics, University of Wollongong, Wollongong, 2522 Australia; 2 CSIRO, Clayton, 3168 Australia; 3 Imaging and Medical Beamline, ANSTO/Australian Synchrotron, Melbourne, 3168 Australia; 4 Department of Radiation Oncology, Prince of Wales Hospital, Randwick, 2031 Australia

**Keywords:** Radiotherapy, Software, Applied physics

## Abstract

Microbeam Radiation Therapy (MRT) is an emerging cancer treatment modality characterised by the use of high-intensity synchrotron-generated x-rays, spatially fractionated by a multi-slit collimator (MSC), to ablate target tumours. The implementation of an accurate treatment planning system, coupled with simulation tools that allow for independent verification of calculated dose distributions are required to ensure optimal treatment outcomes via reliable dose delivery. In this article we present data from the first Geant4 Monte Carlo radiation transport model of the Imaging and Medical Beamline at the Australian Synchrotron. We have developed the model for use as an independent verification tool for experiments in one of three MRT delivery rooms and therefore compare simulation results with equivalent experimental data. The normalised x-ray spectra produced by the Geant4 model and a previously validated analytical model, SPEC, showed very good agreement using wiggler magnetic field strengths of 2 and 3 T. However, the validity of absolute photon flux at the plane of the Phase Space File (PSF) for a fixed number of simulated electrons was unable to be established. This work shows a possible limitation of the *G*4*SynchrotronRadiation* process to model synchrotron radiation when using a variable magnetic field. To account for this limitation, experimentally derived normalisation factors for each wiggler field strength determined under reference conditions were implemented. Experimentally measured broadbeam and microbeam dose distributions within a Gammex RMI457 Solid Water^®^ phantom were compared to simulated distributions generated by the Geant4 model. Simulated and measured broadbeam dose distributions agreed within 3% for all investigated configurations and measured depths. Agreement between the simulated and measured microbeam dose distributions agreed within 5% for all investigated configurations and measured depths.

## Introduction

The objective of radiotherapy is to deliver high doses of radiation to the tumour to ensure local tumour control while simultaneously minimising normal tissue toxicity complications. In current clinical practice it is impossible to entirely spare the normal tissue during treatment delivery. Improvements in conventional techniques to minimise the doses delivered to normal tissues have seen improvements in patient outcomes^1–6^; however, normal tissue toxicity is a limiting factor in efficacy of radio-resistant cancer treatment. The constraints to dose delivery by conventional techniques so as to avoid adverse effects presented by normal tissues remains one of the primary motivational factors to develop novel radiotherapy delivery techniques and modalities^7–9^. The treatment of radio-resistant cancers using Microbeam Radiation Therapy (MRT) is based on the possibility of improved therapeutic ratios resulting from reduced normal tissue complications while maintaining high tumour control probability when exposed to high, spatially fractionated doses. Whilst MRT is currently in the pre-clinical stage, there are many studies demonstrating the efficiency of the MRT treatment modality^10–18^. The underlying physical, chemical, and biological mechanisms that govern the positive selectivity observed between the normal and cancerous tissue response in MRT are yet to be completely understood. Precise treatment delivery models play an essential role in expediting the increase in this understanding and facilitating future human clinical trials.

Three MRT delivery rooms (commonly referred to as hutches) are available for use on the Imaging and Medical beamline (IMBL) at the ANSTO/Australian Synchrotron (AS). At the AS, MRT is delivered via a brilliant, synchrotron-generated x-ray beam, with a photon spectral energy range extending from 40–300 keV^19,20^, which is highly polarised in the electron orbital plane^21,22^. The use of a synchrotron coupled to a dedicated multistage bending magnet or ‘wiggler’ to generate x-rays results in a treatment beam with very small beam divergence and a much higher dose rate in comparison with conventional clinical x-ray treatment beams. For MRT the x-ray beam (denoted as the broadbeam [BB]) is collimated by a multi-slit collimator (MSC), typically made of tungsten carbide, producing the array of x-ray microbeams (MBs) used as a conduit to deliver a therapeutic treatment dose to a cancer target. High dose-rate ‘peaks’ within the microbeam (MB) array are 50 μm wide, and separated by low dose-rate ‘valleys’, with a pitch of 400 μm. The MRT beam features are designed to allow treatment delivery that minimises the impact of patient/organ motion on the broadening of the MRT peak dose, so as to preserve the corresponding spatial fractionation of the MRT treatment dose field. In contrast to the millimeter-sized spatial resolution implemented in conventional radiotherapy, MRT ideally requires micrometer-sized spatial resolution to enable adequate sampling of the MB peaks. This spatial resolution requirement leads to significantly increased complexity to attain the desired signal-to-noise ratio in the clinically relevant dosimetric quantities of peak dose, valley dose (between MBs), peak to valley dose ratio (PVDR), and full width at half-maximum (FWHM) of the MBs. Experimental measurements of dosimetric quantities of interest may be compared to theoretically calculated values to provide quality assurance of the radiation field. Monte Carlo (MC) radiation transport simulations are based on the use of pseudorandom number generation to approximate the stochastic nature of radiation interactions. MC radiation transport simulations require large computational times and associated resources for accurate analysis of complicated radiation field geometries. The long computational times are a limiting factor of MC simulations for MRT. As such, simplified MC methods^10,23–29^ and analytical techniques are usually implemented in treatment planning systems (TPS)^30–32^. However, it is imperative for patient safety that MC codes are used for independent patient-specific pre-treatment verification of dose distributions obtained by the TPS^33^. IAEA Technical Reports Series No. 430^34^ indicates the need for dose verification of the simplified models implemented by TPS in order to mitigate the risk of dose calculation errors.

In this article we describe the development and validation of a full Geant4-based MC model of the IMBL at the AS for wiggler magnetic field strengths of 2 and 3 T. Geant4 is a C++ Object-Oriented MC toolkit that describes particle interactions and transport in matter, originally developed for high energy physics, that has since found extensive use in the fields of radiotherapy, radiation protection, and medical imaging^35,36^. Validation of the model was divided into two sections: (i) Comparison of the Geant4-generated x-ray spectrum against a previously validated analytical model and (ii) Comparison of simulated BB and MB dose distributions within a RMI457 Gammex Solid Water^®^ phantom compared to experimental PTW PinPoint® (model 31014) ionisation chamber (IC) and Gafchromic™ EBT3 film measurements.

Geant4 provides the essential features needed to satisfy the requirements of MRT simulations: the ability to model pseudo time-dependent geometries required for dose delivery in MRT (due to the stationary beam requiring vertical patient translation); a selection of photon and electron physics models both with and without polarisation that have been validated for common materials in the energy ranges relevant for radiotherapy applications^37,38^; the ability to model complex geometries via a computer-aided design (CAD) interface^39^; and the ability to import data using the DICOM interface to read-in patient-specific geometries acquired by CT scans^40,41^.

This article is an important first step towards a full Geant4-based IMBL MRT model for independent, precise, patient-specific, pre-treatment verification of dose distributions delivered using this emerging cancer treatment modality. In conjunction with quality assurance dosimeters and in-beam monitors, this will form an integral part of the accurate and safe delivery of MRT at the AS.

## Materials and Methods

The ability to accurately and efficiently predict the distribution of delivered dose to the patient based on the planned treatment parameters is vital to patient safety in any radiation treatment modality. The accuracy of MC simulations makes them ideal for treatment plan verification and for development and optimisation of novel radiation detectors used for quality assurance^42–48^. The development of the Geant4 MC model for the IMBL at the AS is based upon a previously benchmarked Geant4 application describing the ID17 Biomedical beamline at European Synchrotron Radiation Facility (ESRF)^49^ interfaced with SHADOW x-ray optics and ray-tracing libraries^50^ to model the production of synchrotron radiation in the wiggler magnets. When developing the Geant4 application for IMBL this dependency was removed and the whole beamline, including the wiggler, was modelled in Geant4.

**Figure 1:**
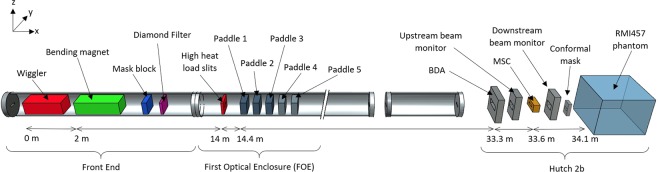
Schematic of the IMBL beamline modelled in Geant4.

Geant4 MC toolkit version 9.6 patch 4 was used in the simulation. The simulation is carried out in two stages: (i) production and transportation of photons to the phantom, (ii) energy deposition within a voxelised phantom geometry. Stage One models the synchrotron radiation production in the insertion device and transports the resulting photons through the IMBL MRT beamline where they are then stored in a phase-space file (PSF) prior to the phantom for use in Stage Two. Stage Two consists of high spatial resolution dose calculations in the vertically scanned phantom, (see Fig. 1). The splitting of the simulation into two distinct phases allows re-use of the same radiation field, stored as a PSF, to calculate the dose in different phantom conditions for the same beamline configuration without rerunning Stage One. A library of PSFs generated using the most common beamline configurations used in treatment would make Stage I simulations unnecessary for day-to-day patient-specific dose verification, thus significantly reducing required simulation time per patient.

The simulations were performed on the M2 MASSIVE cluster^51^. This cluster consists of 42 nodes with 12 cores per node running at 2.66 GHz. Each node has 48 GB of RAM and 2 nVidia M2070 GPUs with 6 GB of DDR5 RAM. The typical PSFs used for MB and BB dose delivery contained 12 × 10^6^ and 21 × 10^6^ unique photons respectively.

For all simulations used in this study 100 separate jobs were submitted on either a single cluster node or split between separate nodes. Each job is assigned a unique seed for its respective random number generator. The mean simulated dose across all jobs was calculated within each voxel with uncertainties represented by a standard error of the mean with a 95% confidence limit. The photon spectra in the PSFs for each of the simulated BDA configurations and maximum wiggler field strengths were analysed and compared to the previously validated analytical model, SPEC^20^.

### Physics

The Low Energy Livermore Polarized Physics list^52^ was activated for this simulation. This physics list models the photoelectric, Rayleigh, and Compton interactions for photons and is valid for an energy range between approximately 250 eV and 10 GeV. Multiple Coulomb scattering, ionisation, and Bremsstrahlung production are modelled for electrons. The photon cross sections have been validated over the energy range between 1 keV and 100 GeV^36^.

The generation of synchrotron radiation is handled by the *G*4*SynchrotronRadiation* process. For this simulation the maximum step-length in the wiggler is set to 0.1 mm and the minimum energy of synchrotron photon production is set to 40 keV. The minimum energy is set to this level to avoid transporting very low energy photons that will be fully absorbed before they reach the phantom. As electrons traverse the length of the wiggler synchrotron photons are produced with the *G*4*SynchrotronRadiation* process^53^ using the *G*4*HelixSimpleRunge* solver to approximate the curved path of the electron in the magnetic field. For details the reader is directed to the Geant4 Physics Reference Manual^52^.

In order to increase the simulation efficiency a global ‘production cut’ of 0.01 mm was adopted. The ‘cut’ was optimised to reduce simulation times whilst maintaining a adequate degree of accuracy in the dosimetric results of the simulation.

### Geant4 simulation stage one - synchrotron radiation production

The wiggler parameters used for synchrotron radiation production are outlined in Table 1. Following the synchrotron radiation production in the wiggler the electrons are directed into a ‘kill plane’ by the bending magnet positioned after the wiggler, 3 m from the source. The kill plane stops the transportation of the electrons to speed up the simulation times as they are no longer required after this point in the simulation. The photons are transported through the IMBL MRT beamline, as shown in Fig. 1. On the IMBL interchangeable tungsten beam defining apertures (BDAs) provide intrinsic x-ray radiation beam dimensions of constant 30 mm width (Y) and variable height (Z) of 0.532, 1.053, or 2.014 mm. The addition of tungsten conformal masks positioned approximately 0.5 m downstream from the BDA allow for the further reduction of the intrinsic beam width to 5, 10 or 20 mm. A synchrotron-generated BB can also be utilised when the MSC is removed and the intrinsic field is only defined by the BDA and conformal mask for a more conventional flat beam profile. The beam heights are defined using the BDAs to provide consistent and reproducible beam dimensions. Full details of the beamline component positions have been provided by Stevenson *et al*.^20^ and so will not be repeated here. However, details on filtration configurations used in this work can be found in Table 1.

**Table 1:** Beamline parameters used for synchrotron photon generation where; *I*_*ring*_ is the beamline ring current in continuous top-up mode, *E*_*ring*_ is the energy of electrons within the ring, *B*_*peak*_ is the peak wiggler field, *λ* is the period of the wiggler field, *N*_*periods*_ is the number of periods of the wiggler field, and *D*_*source*_ is the magnetic length of the wiggler.

Machine Parameters		
*I*_*ring*_	200.1 mA	
*E*_*ring*_	3.032 GeV	
**Wiggler Parameters**		
*B*_*peak*_	2 and 3 T	
*λ*	52 mm	
*N*_*periods*_	30	
*D*_*source*_	1.56 m	
**Beamline Filtration**		
	**3** **T filtration**	**2** **T filtration**
Paddle 1	0.45 mm graphene at 90°	0.45 mm graphene at 90°
Paddle 2	+5 mm HD graphite at 45° (7.07 mm)	+5 mm HD graphite at 45° (7.07 mm)
Paddle 3	+10 mm HD graphite at 45° (14.14 mm)	+10 mm HD graphite at 45° (14.14 mm)
Paddle 4	+1 mm Cu at 45° (1.41 mm)	+1 mm Cu at 45° (1.41 mm)
Paddle 5	+1 mm Cu at 45° (1.41 mm)	+2 mm Al at 45° (2.83 mm)

After generation and transportation of the photons through the beamline each photon that crosses the phase-space scoring plane has its position, momentum, polarisation, and energy recorded in the PSF. The phase-space scoring plane is positioned in Hutch 2B at a distance of 33.4 m from the source (downstream from the BDA), just after the MSC. This allows for the same position of the PSF to be maintained in both the BB and MB configurations.

### Stage two - dose delivery in user specified phantom

Stage two of the simulation consists of dose calculations in the vertically scanned phantom incorporating the target volume, and positioned at a distance 34.16 m from the source. As aforementioned, the vertical height of the synchrotron generated x-rays is limited by the BDAs creating an effective intrinsic treatment beam height. In order to treat targets of heights greater than 2 mm the target is moved vertically at a constant speed to ensure full coverage of the target. The equivalent “dose painting” technique is commonly used in charged particle therapy by steering a particle pencil beam. The movement of the phantom stage in the z-direction is simulated by Geant4 using a time-dependent parameterised geometry^54^, where the vertical translation step size of the phantom is determined by dividing the desired field length by the total number of primary particles to be simulated. For experimental data acquisition the sample stage was vertically translated at a constant vertical velocity of 10 mm·s^−1^ through the beam in a single scan.

For BB simulations the phantom was modelled as a rectangular slab with dimensions 140 mm × 100 mm × 100 mm (X,Y,Z). The material of the phantom was modelled as Gammex RMI457 Solid Water^®^ to match the experimental setup. Gammex RMI457 Solid Water^®^ was chosen for this validation study as the dose delivered within this material has been shown to be equivalent to water in the photon energy range of interest for MRT^38^. A voxelised scoring mesh with dimensions 140 mm × 100 mm × 100 mm was overlayed on the modelled rectangular phantom. This mesh recorded the spatial distribution of energy deposition within the phantom, allowing a determination of the dose delivered. A voxel size of 2 mm × 2 mm × 5 mm for the BB configuration was used. This voxel size was chosen to approximate the size of the PTW PinPoint® Ionisation Chamber (IC) sensitive volume used in the experiments. For the MB configuration voxel sizes of 1 mm × 0.01 mm × 0.1 mm were used to reduce simulation time whilst maintaining adequate spatial resolution to resolve the MB profile. Given the increased complexity of the MB simulations and decreased voxel size, the dimensions of the voxelised scoring mesh was reduced to a 140 mm × 2 mm × 2 mm volume placed centrally within the phantom.

### Dose delivery validation

Experimental validation of the dose delivery was carried out on both BB and MB configurations. The BB configuration chosen in the validation for comparison with the PTW PinPoint® IC was 20 mm × 20 mm (Y,Z), since the chamber is only recommended for field sizes of 20 mm × 20 mm or larger. BDA heights of 1.053, and 2.014 mm were used for MB dose delivery. The BB and MB x-ray treatment field height and width for each configuration was kept at a constant 20 mm and was defined by the conformal mask that moves with the target volume, with the target volume centred with respect to the mask along the line of the x-ray beam.

Under experimental conditions the phantom is translated in the Z-direction at constant speed so the intrinsic synchrotron x-ray beam is effectively incident on the phantom for a known time (beam-on time) depending on the treatment field size and the calculated speed. The calculated speed, *ν*_*z*_, depends on the desired dose, *D*, intrinsic dose rate, $\dot{D}$, and BDA height, *h* as shown in Eq. 1. In the second MRT treatment room of the IMBL at the AS, the recommended fastest speed used is 10 mm·s^−1^; so, for example, using the 20 mm × 20 mm mask leads to an effective beam-on time of 2 seconds. In order to simulate a beam-on time of 2 seconds using Geant4, for a maximum wiggler field strength of 3 T and a BDA of 2 mm height, running 100 jobs on 100 separate nodes would take approximately 10^21^ hours to produce an x-ray photon distribution, comparable to that produced by SPEC. Whilst the chosen approach has the disadvantage of increased computational time, it should be noted that it provides a clear benefit in terms of particle information, i.e. momentum, polarisation etc, which is not given by SPEC. Due to the unrealistic simulation time required, the calculated dose delivered within the phantom using Geant4 is scaled to the actual experimental conditions according to the ratio of the storage ring current in the experiment and the number of simulated electrons entering the wiggler, as shown in Eq. 2.

1 $${\nu }_{z}=\frac{\dot{D}\times h}{D}$$

2 $$D=\frac{{E}_{v}}{{m}_{v}}\times \frac{{I}_{ring}\times {t}_{\exp }}{{n}_{{e}^{-}sim}}$$

where D = dose (Gy), *E*_*v*_ = energy deposited in voxel (keV), *m*_*v*_ = mass of voxel (kg), *I*_*ring*_ = current in the storage ring, *t*_*exp*_ = experimental beam-on time and *n*_*e*−*sim*_ = number of simulated electrons.

For the validation of the BB configuration, calculations were compared to dosimetric profiles measured using the PTW PinPoint® IC and Gafchromic™ EBT3 film. Due to the limitations in the sensitive volume size the IC is inadequate for use in the MB configuration. As such only EBT3 film are able to be used for the MB irradiation configuration. In order to reduce the film uncertainties relevant planned treatment doses are ideally selected (in both BB and MB configurations at the phantom depths measured) to ensure that all doses fall within the limited operating range of the film. This is more challenging in MB irradiations than for BB due to the significant differences in the peak dose and the valley dose.

The EBT3 film was scanned using a Leica (Leica Microsystems, Wetzlar, Germany) DMI4000B microscope with an 8-bit Leica DMC2900 camera coupled to a 10x objective lens. For each film the scanning procedure consisted of multiple scans using the motorised sample stage which were stitched together using the Leica Application Systems software, producing a single 8-bit TIFF image. A total of 30 scans per film were implemented to produce a final film scan with dimensions of 1.5 cm × 1.5 cm positioned at the centre of the 2 cm × 2 cm film. Each film was scanned both prior and post-irradiation, allowing a 24 hour development time before post-irradiation scans were taken as a trade off between film development stability and experimental practicality (strict time frame limits for synchrotron facility experiments).

To minimise the influence of external light on the film scans the microscope is housed in a large volume incubator with an opaque covering. For each scan a white field correction was applied to correct for radial non-uniformity of the film scan caused by the microscope light.

The average pixel value for the calibration and BB film scans was determined using a 1 cm diameter region of interest positioned at the centre of each film scan. For the MB film scans the analysis procedure consisted of a 1 cm × 1 cm (Y, Z) rectangular region of interest positioned at the centre of the film. The 1D Y-profile was then determined by averaging the pixel values over the Z axis for each Y position in 1 μm steps. This MB analysis procedure was performed for both the film and simulation measurements to ensure parity between the two. The profiles measured by the film were extracted for discrete depths of interest within the phantom and compared to the simulation results.

The pixel value was determined using single-channel analysis and correcting the post-irradiation scan by the pre-irradiation scan for each film, see Eq. 3.

3 $${P}_{Corrected}=|{P}_{Post}-{P}_{{\Pr }e}|$$

where *P*_*Post*_ = pixel value post-irradiation, *P*_*Pre*_ = pixel value pre-irradiation and *P*_*Corrected*_ = corrected pixel value.

The green channel was chosen for this work using the read-out and scanning strategies developed for the Leica microscope imaging system. Compared to using the red channel for analysis it provided a larger dynamic range while maintaining comparable errors. The blue channel was deemed unsuitable for this work due to the lack of reproducibility in measurements coupled with poor sensitivity over the range of interest.

The recommendation from the manufacturer of the film is to scan the film at 72 dpi; however, the scanning resolution of approximately 3600 dpi was implemented. Scanning at this resolution resulted in one pixel per seven micrometres and allowed for adequate resolution to resolve the microbeams. A caveat of scanning with this resolution is that the ‘granular’ structure of the film is apparent and may produce image artefacts. The effect of the granular structure on the corrected pixel value is a greater minimum pixel value and reduction in film scan uniformity.

The overall uncertainty of the Gafchromic™ film dosimetry system used in this work for the green channel was calculated using the ISO Guide to the expression of Uncertainty in Measurement (GUM) methodology^55^, the overview of the uncertainties are shown in Table 2.

**Table 2:** Overall uncertainty of film dosimetry protocol using single channel analysis for MRT implemented in this work.

Source	Type A	Type B
Film Scan Uniformity	3.6	
Microscope Light Influence	1.3	
Positioning Reproducibility	0.4	
Signal Growth*		2.1
Sheet Uniformity	0.6	
Reproducibility	2.7	
Combined Standard Uncertainty in Pixel Value (%)	5.2	
Expanded Uncertainty in Pixel Value (%)	10.4**	

*Signal growth error based off a development time of (24 ± 2) hours. **Expanded uncertainty based on the standard uncertainty multiplied by a coverage factor of k = 2, providing a coverage probability of approximately 95%.

**Figure 2:**
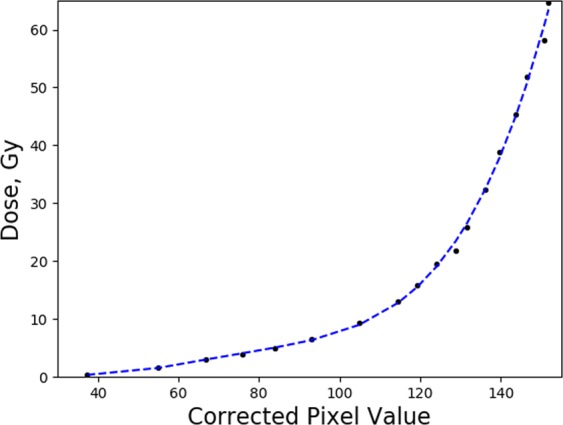
Calibration curve using green channel analysis for EBT3 film implemented in this work. Polynomial fit given in Eq. 4 depicted by the blue dotted curve.

The same scanning and read-out procedure was followed for the calibration films. Each of the calibration films were irradiated at a constant depth of 2 cm in a Gammex RMI457 Solid Water^®^ phantom. The literature has demonstrated the ability to extend the useful range of EBT3 well above the manufacture’s recommended range using an array of different scanning techniques^56–58^. The scanning technique implemented in this work allowed for absorbed doses of up to 65 Gy to be delivered to the EBT3 film, provided an adequate number of calibration points were chosen. The absolute limits of the methodology implemented in this work are currently under investigation. The calibration curve implemented for this work is shown in Fig. 2, for the dose range of 0.25–64 Gy and associated polynomial fit depicted in Eq. 4. The total dose delivered to the film was adjusted by varying the speed of the motorised sample stage, see Eq. 1.

4 $$Dose=a\times {P}_{Corrected}^{4}+b\times {P}_{Corrected}^{3}+c\times {P}_{Corrected}^{2}+d\times {P}_{Corrected}+e$$

where a = 1.15 × 10^−6^, b = −3.21 × 10^−4^, c = 3.33 × 10^−2^, d = −1.40 and e = 20.5.

The dose delivered to the PTW PinPoint® IC was read out using the PTW Unidos Webline electrometer coupled to a 10 m extension cable. Biasing conditions were set to those specified for the calibration certification supplied by PTW: a bias of +400 V was applied to the IC using the internal biasing function of the electrometer. The following correction factors were applied to the PTW PinPoint® IC throughout this work: temperature and pressure (*k*_*TP*_), polarisation (*k*_*pol*_), electrometer calibration factor (*k*_*elec*_), and recombination (*k*_*s*_). The calculated chamber specific energy correction (*k*_*Q*_), was taken as 0.95, per the calibration certificate.

## Results and Discussion

### Photon spectrum

**Figure 3:**
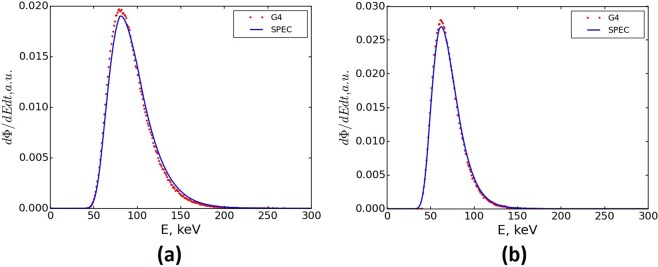
Frequency distribution of the BB x-ray photon spectra from Geant4 compared to SPEC for (**a**) 3 T and (**b**) 2 T wiggler field strengths.

The Geant4 simulated and SPEC calculated photon spectra for two different wiggler magnetic field configurations are displayed in Fig. 3. The average simulation time per-job for the generation of the BB x-ray spectrum with a maximum wiggler field strength of 3 T were 16, 35, and 75 hours for the 2.014 mm, 1.053 mm, and 0.532 mm BDAs respectively. The simulation times for the MB configurations, on average, required 2.5 times longer to produce the same number of photons at the PSF. When the maximum wiggler magnetic field strength was switched from 3 T to 2 T, a decrease in the photon yield was observed as expected. This resulted in approximately 45% longer Stage One execution times for similar sized PSF files. The change in wiggler field or BDA did not impact simulation times for Stage Two of the simulation as this stage is only dependent on the number of photons in the PSF. The average simulation time for Stage Two dose delivery within the phantom for BB and MB were 20 and 40 minutes respectively, for all configurations.

The normalised spectra generated by Geant4 for each intrinsic beam configuration were identical, thus only the 2.014 mm BDA results for each wiggler are shown in Fig. 3. Slight differences are evident between the spectra generated by Geant4 and SPEC in Fig. 3. These differences can be attributed to the underlying calculation models and assumptions between the programs; SPEC using an analytical approach in contrast to Geant4’s MC approach.

The comparison between the normalised photon spectra for wiggler field strengths of 3 and 2 T generated by SPEC and Geant4 at the plane of the PSF are depicted in Table 3. Good agreement between the generated spectra maximum value and mean energy is seen, with a percentage difference between the mean of energy of the generated spectra of 2.92% and 1.70% and percentage difference of spectra maximum values of 2.5% and 0.3% for wiggler field strengths of 3 and 2 T respectively.

**Table 3:** Comparison between normalised photon spectra generated by SPEC and Geant4 at the plane of the PSF.

Spectra Comparison				
Field Strength	3 T		2 T	
Modality	SPEC	Geant4	SPEC	Geant4
Spectra Maximum (keV)	81	79	61.9	62.1
Mean Energy (keV)	94.63	91.91	70.73	69.54
Uncertainty Mean (keV)	0.61	0.01	0.51	0.01
*ϕ*_*SPEC*_/*ϕ*_Geant4_	2		2.6	

Where *ϕ*_*SPEC*_/*ϕ*_Geant4_ is the ratio of absolute number of photons produced by SPEC vs Geant4 for a fixed number of simulated electrons.

However, when comparing the expected and simulated photon fluence for a time interval dt, given a fixed beam current, the Geant4 simulated photon fluence deviated from the expected fluence at the position of the PSF. The ratio of expected photons to Geant4 simulated photons reaching the plane of the PSF for a fixed number of electrons were 2.00 and 2.60 for wiggler field strengths of 3 and 2 T respectively.

The energy distribution of photons in the range of 40–300 keV is in excellent agreement indicated by the close proximity in values of *E*_*max*_ and *E*_*mean*_, however the flux is otherwise. The cause of this deviation may be attributed to the *G*4*SynchrotronRadiation* process which has been validated for constant magnetic fields. This model is a good approximation for photon production in magnetic fields which remain relatively constant over the formation length for synchrotron radiation^52^, however has yet to be validated for varying magnetic fields such as that produced from a multi-pole wiggler. The current implementation of the *G*4*SynchrotronRadiation* process leads to increased production of low energy photons which are preferentially absorbed by the beamline filtration and account for the deviation seen at the plane of the PSF. This may suggest development of a model valid for use in wiggler fields be implemented in future work. Subsequently, the validity with respect to the total number of photons produced as a function of simulated electrons has not been established and is under active investigation. As a result, the Geant4 simulations require normalisation to experimental data and limits the capability of calculating and comparing absolute doses. Equation 2 has been updated to include a calibration factor to account for this discrepancy using the experimental reference conditions data at 2 cm depth for each wiggler field strength, see Eqs. 5 and 6.

5 $$D=\frac{{E}_{v}}{{m}_{v}}\times \frac{{I}_{ring}\times {t}_{\exp }}{{n}_{{e}^{-}sim}}\times {K}_{w}$$

6 $${K}_{w}=\frac{{D}_{\exp ,1{\rm{mm}}}}{{D}_{sim,1mm}}$$

where *K*_*w*_ = calibration value for the simulation as a function of maximum wiggler field strength, *D*_*exp*,1mm_ = experimental dose delivered under reference conditions for the 1.053 mm BDA and *D*_*sim*,1mm_ = simulation dose delivered under reference conditions for the 1.053 mm BDA. The reference dosimetry protocol conditions implemented on IMBL emulate the ESRF protocol^59^, with a measurement depth of 2 cm, a 20 mm × 20 mm field size and a sample stage translation speed of 10 mm·s^-1^. For this work a Gammex RMI457 Solid Water^®^ phantom was used to enable film dosimetry to be performed.

### Geant4 compared to IC and EBT3 film for synchrotron BB dose delivery

The *K*_*w*_ factors determined using the measured dose under reference conditions in Eq. 6 were 1.91 and 2.54 for wiggler field strengths of 3 and 2 T respectively. Table 4 depicts the comparison between the ratio *D*_*exp*_/*D*_*sim*_ for each wiggler field strength and beam configuration at 20 mm depth within the Gammex RMI457 Solid Water^®^ phantom. For each wiggler field strength there is good agreement between all BDA configurations, however the 1.053 mm BDA is chosen as the reference beam geometry due to the improved experimental reproducibility of the beam compared when defined using the 2.014 mm BDA and increased experimental beam stability compared when defined using the 0.532 mm beam.

**Table 4:** Comparison between ratio of *D*_*exp*_/*D*_*sim*_ for each wiggler field strength and beam configuration at 20 mm depth within the Gammex RMI457 Solid Water^®^ phantom, with uncertainties represented by two standard deviations.

Dose Comparison		
BDA height (mm)	*D*_*exp*_/*D*_*sim*_	
	3 T	2 T
2.014	1.95	2.45
1.053	1.91	2.54
0.532	1.94	2.49
Mean	1.93	2.49
Uncertainty	0.04	0.09

The calculated *K*_*w*_ values agreed with the deviation in absolute number of photons produced for a fixed number of simulated electrons (*ϕ*_*SPEC*_/*ϕ*_*Geant*4_) of 2.00 and 2.60 for wiggler field strengths of 3 and 2 T respectively. The percentage difference between the calculated *K*_*w*_ and *ϕ*_*SPEC*_/*ϕ*_*Geant*4_ were 4.6 and 2.3% for wiggler field strengths of 3 and 2 T respectively. This further indicates the *G*4*SynchrotronRadiation* process implemented in this work is producing a correct distribution of photons at the plane of the PSF, however with an incorrect magnitude.

**Figure 4:**
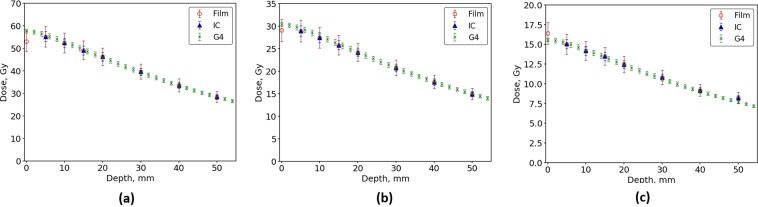
Simulated BB dose as a function of depth in a Gammex RMI457 Solid Water^®^ phantom using Geant4 for a 3 T wiggler field and a BDA of (**a**) 2.014 mm (**b**) 1.053 mm and (**c**) 0.532 mm height. Measured data using a PTW PinPoint® ionisation chamber and EBT3 film are overlayed.

**Figure 5:**
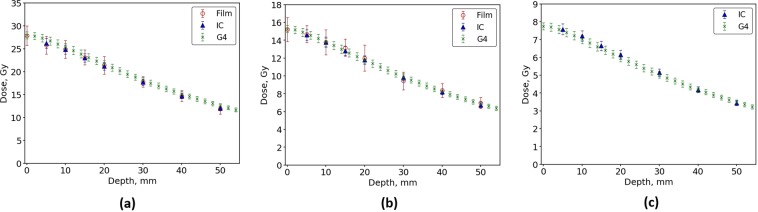
Simulated BB dose as a function of depth in a Gammex RMI457 Solid Water^®^ phantom using Geant4 for a 2 T wiggler field and a BDA of (**a**) 2.014 mm, (**b**) 1.053 mm and (**c**) 0.532 mm height. Measured data using a PTW PinPoint® ionisation chamber and EBT3 film are overlayed for 2.014 mm and 1.053 mm BDA. Only IC data was taken for the 0.532 mm BDA.

Figures 4 and 5 show the dose delivered as a function of depth for a BB treatment field of 20 mm × 20 mm within the Gammex RMI457 Solid Water^®^ phantom. The simulated doses were compared to the PTW PinPoint® IC and EBT3 film for a range of intrinsic x-ray beams of varying height, as defined by the BDA. Overall there was seen to be excellent agreement across all three intrinsic beams tested for the 3 T wiggler and the two intrinsic beams tested for the 2 T wiggler. The IC measurement depths begin from 5 mm depth and compare extremely well with the EBT3 film and simulation data. The EBT3 film calibration was performed at 20 mm depth using the 1.053 mm BDA for all BB and MB measurements.

When using a maximum wiggler magnetic field strength of 3 T, the dose determined by the simulation agrees within 2% across all depths for each BDA configuration. The maximum deviations were seen when using the 2.014 mm BDA to define the intrinsic beam, with an average percentage difference of 1.7%. For the other two BDAs the average percentage difference was found to be 0.9% for the 1.053 BDA and 1.1% for the 0.532 mm BDA. The largest variation between simulated and measured dose, at either wiggler magnetic field, was seen for the EBT3 film data very close to the surface of the phantom. However this variation was within the uncertainty of the measurements and simulations so was not considered significant enough to warrant further investigation.

For each BDA configuration using the 2 T wiggler, the dose delivered with depth in the simulation agrees within 3% across all depths to those measured by the IC and film. The maximum deviations were seen when using the 1.053 mm BDA to define the intrinsic beam, with an average percentage difference of 2.3%. For the 2.014 mm BDA the average percentage difference across all depths was found to be 1.6% and 1.4% for the 0.532 mm BDA.

### Geant4 compared to EBT3 film for synchrotron MB dose delivery

**Figure 6:**
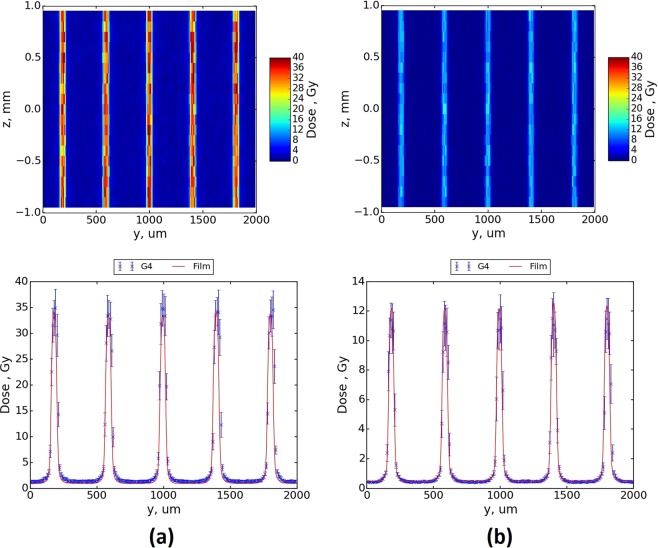
Two dimensional dose distribution (top) and 1D dose profiles (bottom) at 2 cm depth within a RMI457 Solid Water^®^ phantom for (**a**) BDA of height 2.014 mm and (**b**) BDA of height 1.053 mm, with a maximum wiggler field strength of 3 T.

Figure 6 shows a comparison between simulated and measured MB profiles for the five central microbeams using the (a) 2.014 mm and (b) 1.053 mm BDAs. The depth for the comparison is 20 mm in the Gammex RMI457 Solid Water^®^ phantom, the wiggler field strength is 3 T and the target translation speed is 10 mm·s^−1^. The top plot in Fig. 6 is a colour map of the simulated dose near the centre of the MRT treatment field (target centre is at 0, 0 of the *y* − *z* plane and x, the depth, is 20 mm. In the bottom plots of Fig. 6 the 2D profile of the central five microbeams for the simulated MB (in the plots above) are compared with the equivalent measured MB profiles. Overall, one can see there is very good agreement between the simulated and measured plots, however it is immediately clear from the 2D profile plots in Fig. 6 that the uncertainties in the simulated data could obviously be reduced by further simulation data. Such data would require an increase in the overall simulation time required above the current 23 minutes. In any case, the comparison is limited by the reasonably achievable uncertainty in the film data, and is discussed in more detail below. The error bars in the film data in Fig. 6 have therefore been removed to better illustrate the comparison, refer to Table 2 for the overall film errors associated with the measurements.

In comparing Geant4 simulation data to the EBT3 measurement data for Fig. 6, a full dataset for the 3 T wiggler field with the intrinsic beams defined by the 2.014 mm and 1.053 mm BDAs was able to be completed. However, no such comparison could be made for the 0.532 mm BDA. Further to this, the equivalent 2 T wiggler field case dataset was even more restricted, and only the peak doses using the 2.014 mm and 1.053 mm BDAs were able to be compared. The restricted datasets are due to the combined effect of the limited dynamic range of the film, ‘granular’ structure of the film, and the inability to deliver doses less than 0.25 Gy for calibration purposes. The dose delivery limitation for calibration is because in the BB configuration the air kerma rate is approximately 1 kGy·s^−1^ and with the current motor and BDA configurations available on the IMBL at AS, delivery of doses below 0.25 Gy is not feasible. As such, valley doses for the MB treatment delivery when using the EBT3 film are unreliable when they are below the minimum dose used for the calibration of the film. This was seen for all 2 T results as well as the 3 T results using the 0.532 mm for between 80 and 140 mm depth. The valley doses were omitted for the configurations where they could not be reliably calculated from the film data. Peak values as a function of depth were compared for these configurations. In future either the two-film technique or the use of Gafchromic™ HDV2 film may be employed. The two-film technique requires a primary film to be used to measure the peak and a secondary film to measure the valley, subsequently enabling potentially more reliable measurements of both the peak and the valley dose. However, the strict spatial resolution requirement for the peak dose estimates and the associated resulting uncertainties will remain a challenge. The implementation of HDV2 film, with a larger dynamic range, will allow for higher doses to be delivered to the film. Simultaneous peak and valley doses measurements using one film will present the same challenges as seen for EBT3.

The larger voxel size of 10 μm used in the simulation compared to the smaller sensitive volume of approximately 7 μm of the EBT3 film results in notably sharper shoulders in the EBT3 profiles. Good agreement was seen across the profiles for the 2.014 mm and 1.053 mm BDAs, with the mean percentage difference in the peak values of 1.4% and 3.2% respectively. Good agreement was seen for the valley dose values above the film dose threshold, with a mean percentage difference of 1.3% and 0.93% respectively. Agreement in the shoulders of the profiles can be improved by reducing the size of voxels used in the simulation in the *y* dimension from 10 μm to 1 μm; however, this would result in much slower simulation times as well as larger output file sizes and so remains a challenge for very precise MRT dosimetry.

**Figure 7:**
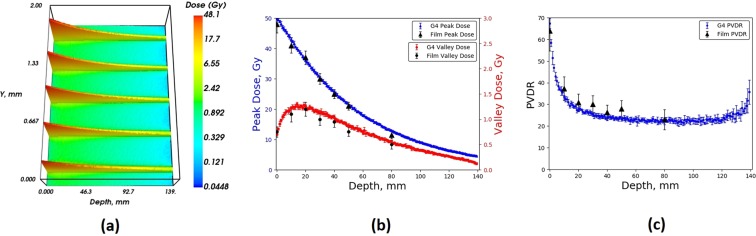
Geant4 (**a**) 2D MB depth dose distribution, (**b**) Peak dose and valley dose, (**c**) PVDR, within an RMI457 Solid Water^®^ phantom for a BDA of height 2.014 mm compared with EBT3 film, for a maximum wiggler field strength of 3 T.

**Figure 8:**
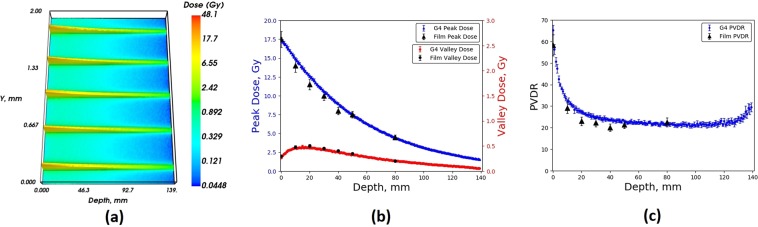
Geant4 (**a**) 2D MB depth dose distribution, (**b**) Peak dose and valley dose, (**c**) PVDR, within an RMI457 Solid Water^®^ phantom for a BDA of height 1.053 mm compared with EBT3 film, simulation for a maximum wiggler field strength of 3 T.

Figure 7(a) and 8(a) show the full 2D depth-dose distribution for the 3 T wiggler field, calculated by the Geant4 simulation, and showing the central five microbeams within the RMI457 Solid Water^®^ phantom as a function of depth using the 2.014 mm and 1.053 mm BDA respectively. The figures are excellent quantitative illustrations of the spatially fractionated MRT irradiation treatment field. The extremely small x-ray source divergence gives rise to the microscopic MBs acting as conduits to the full treatment dose coverage of a macroscopic target volume, which in this case has a cross-sectional area of 20 mm × 20 mm. The short range of the secondary particles associated with MRT ensure the striated structure of the radiation dose field remains very tight with depth and is a unique feature of this exciting emerging cancer treatment modality. Such a tightly striated dose structure with depth is therefore impossible to achieve using conventional clinical LINAC beams or particle therapy beams.

Also shown in Figs. 7 and 8 are summary plots of all of the simulated and measured data combined, displaying the peak dose, valley dose and peak-to-valley dose ratio (PVDR) with depth in the Gammex RMI457 Solid Water^®^ phantom. For each of the 2D depth-dose distributions the average peak and valley was determined as a function of depth within the phantom and compared to EBT3 film. For the 2.014 mm BDA there was good agreement across all depths for the peak, valley and subsequent PVDR values. The average percentage difference for the peak and valley values was found to be 1.8% and 3.7% respectively across all depths with a maximum difference of 2.1% and 4.7% respectively. In regards to the 1.053 mm BDA, the valley dose for the depth of 80 mm was lower than the smallest calibrated dose of 0.25 Gy, and subsequently was omitted from the results shown in Fig. 8.

Figures 7 and 8 demonstrate a reduction in valley dose at the entrance and exit of the phantom. The reduced valley dose in the first 20 mm is a direct result of the lack of incident photons between the MBs (by design) as well as the associated reduced forward scattered photons into the valley regions from the MB peaks due to the low-density air (relative to water) in front of the phantom. The reduced dose at the exit of the phantom is a direct result of the lack of density (relative to water) in the backscatter material (being air again).

**Figure 9:**
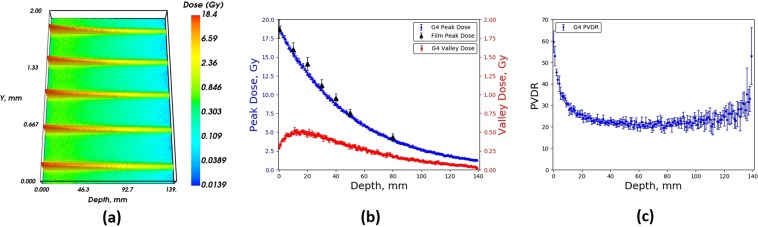
Geant4 (**a**) 2D MB depth dose distribution, (**b**) Peak dose and valley dose, (**c**) PVDR, within an RMI457 Solid Water^®^ phantom for a BDA of height 2.014 mm compared with EBT3 film, simulation for a maximum wiggler field strength of 2 T.

**Figure 10:**
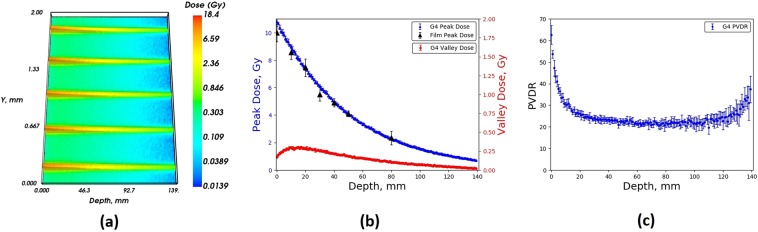
Geant4 (**a**) 2D MB depth dose distribution, (**b**) Peak dose and valley dose, (**c**) PVDR, within an RMI457 Solid Water^®^ phantom for a BDA of height 1.053 mm compared with EBT3 film, simulation for a maximum wiggler field strength of 2 T.

Figures 9 and 10 show the simulated and measured (where possible) peak and valley doses delivered to the Gammex RMI457 Solid Water^®^ phantom as a function of depth for a 2 T wiggler field, for the 2.014 mm and 1.053 mm BDA. Only the peak doses are displayed for the EBT3 film data as the valley doses delivered to the film were below the minimum calibration value of 0.25 Gy. This was the case for all depths within the phantom, hence they are not displayed. Very good agreement is seen between the simulated and measured data. The data set is limited compared to that shown in Figs. 7 and 8 however it represents an extremely important milestone in terms of the MRT beamline model as it is associated with a different wiggler magnetic field which leads to a completely different photon energy spectrum (as seen in Fig. 3). In addition, the angular distribution of the x-ray photons emitted from the IMBL wiggler source is also distinctly different, along with the transmission and scattering probabilities of this x-ray beam through the various filters, windows and MSC, along the 33.4 m distance from the source to the RMI457 phantom.

For the 2.014 mm BDA there is good agreement across all depths for the peak values. The average percentage difference for the peak values was found to be 2.7% across all depths with a maximum difference of 3.4%. Similarly for the 1.053 mm BDA, good agreement is seen between the peak doses measured using the film compared to those determined in the simulation. The average percentage difference for the peak values was found to be 0.4% across all depths with a maximum difference of 0.9%. The better agreement for the 1.053 mm BDA compared to the 2.014 mm BDA may be related to some limitation in the model of the x-ray intensity roll-off or energy roll-off (effectively softening the energy spectrum) with solid angle projected from the wiggler x-ray source in the y-z plane (see Fig. 1). Such an investigation is beyond the scope of this work, but important, and will be the focus of future research in this area. The intensity roll-off is less important experimentally for the 1.053 mm BDA since the intrinsic field size is substantially smaller than the maximum intrinsic field size in the *z*–direction on the IMBL at the AS.

## Conclusion

The first Geant4-based model of the Australian Synchrotron Imaging and Medical Beam Line has been successfully developed and validated in both BB and MB configurations using maximum wiggler magnetic field strengths of 3 and 2 T. Good agreement was seen between the normalised Geant4 simulation-generated photon energy distribution and the previously validated SPEC software. The validity of absolute photon flux at the plane of the PSF for a fixed number of simulated electrons was unable to be established. This work shows a possible limitation of the *G*4*SynchrotronRadiation* process to model synchrotron radiation when using a variable magnetic field, a subject of on-going investigations.

To account for this issue, the use of an experimentally derived normalisation factor, *K*_*w*_, was implemented using the experimental reference conditions for each wiggler field strength. *K*_*w*_ was determined to be 1.91 and 2.54 for wiggler field strengths of 3 and 2 T respectively. The calculated *K*_*w*_ values agreed with the deviation in absolute number of photons produced for a fixed number of simulated electrons of 2.00 and 2.60 for wiggler field strengths of 3 and 2 T respectively.

Excellent agreement was seen in both the BB and MB dose delivered for a range of beam configurations and depths within a Gammex RMI457 Solid Water^®^ phantom. In the BB case the calculated dose agreed within 3% across all depths for maximum wiggler field strengths of 2 and 3 T. The simulated MB dose profiles at 20 mm depth agreed with measured film data obtained under the same conditions within 4%.

Future work aims at validating the Geant4 model for the remaining beamline configurations available on the IMBL. In addition, further development of the MRT treatment simulations to include more complex and realistic treatment geometries for implementation in dosimetric studies for upcoming *in*–*vivo* pre-clinical trials has commenced.
